# Regulating Intratumoral Fungi With Hydrogels: A Novel Approach to Modulating the Tumor Microbiome for Cancer Therapy

**DOI:** 10.1002/cam4.70900

**Published:** 2025-04-30

**Authors:** Ping Chen, Weiwei Tian, Anqi Zeng, Huan Gu, Jin Zeng

**Affiliations:** ^1^ Translational Chinese Medicine Key Laboratory of Sichuan Province Sichuan‐Chongqing Joint Key Laboratory of Innovation of New Drugs of Traditional Chinese Medicine Sichuan Academy of Chinese Medicine Sciences Chengdu China; ^2^ College of Pharmacy and Food Southwest Minzu University Chengdu China

**Keywords:** hydrogels, intra‐tumoral fungal, tumor microbe microenvironment

## Abstract

**Background:**

Fungi in tumors act as a double‐edged sword, potentially worsening or alleviating malignancy based on the ecological balance within the tumor microenvironment (TME). Hydrogels, as innovative drug delivery systems, are poised to redefine treatment paradigms. As advanced biomaterials, they offer a versatile platform for encapsulating and releasing antifungal agents and immunomodulators, responding to the TME's unique demands.

**Methods:**

We have conducted and collated numerous relevant reviews and studies in recent years from three aspects: Hydrogels, intra‐tumoral fungi, and tumor microbe microenvironment, in the hope of identifying the connections between hydrogels and intra‐tumoral microbes.

**Results:**

This review underscores the crucial role of intra‐tumoral microbes, particularly fungi, in tumorigenesis, progression, and treatment efficacy. At the same time, we concentrated on the findings of hydrogels investigations, with their remarkable adaptability to the tumor microenvironment emerge as intelligent drug delivery systems.

**Conclusions:**

Hydrogels unique ability to precisely target and modulate the tumor microflora, including fungi, endows them with a significant edge in enhancing treatment efficacy. This innovative approach not only holds great promise for improving cancer therapy outcomes but also paves the way for developing novel strategies to control metastasis and prevent cancer recurrence.

## Graphical abstract

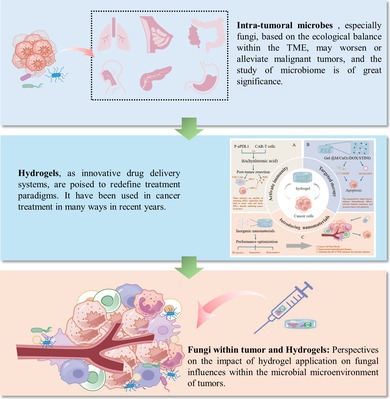

## 1 Introduction

Cancer is one of the leading causes of death from disease globally, and despite the vast amount of research in cancer treatment in recent years, it remains one of the greatest threats to human health. Studies have shown that one of the obvious characteristics of tumor growth is the generation of disorganized and nonfunctional blood vessels, which prevents the drugs from reaching the tumor site smoothly through the bloodstream. Not only is less drug targeted to reach the tumor, but also the residual circulating drug may harm normal tissues and may even promote the invasion and metastasis of the tumor after long‐term use of the drug [1]. To overcome these difficulties, it is particularly important to study the mechanism of tumor development, new drug delivery materials, and drug delivery routes.

According to statistics, there are about trillions of bacteria in the human body that are involved in the regulation of physiological processes and diseases in the organism. In addition to bacteria, human microorganisms include eukaryotes, fungi, prokaryotes, and viruses [2, 3, 4, 5]. The microbiota plays important roles in human health, including regulation of the immune system, action of the digestive system, influencing host metabolism, and influencing the prevention and treatment of disease [6, 7, 8]. Understanding the relationship between microorganisms in the human body is important for maintaining health and preventing and treating diseases. However, research in this area is still evolving, and many mechanisms and roles have yet to be fully elucidated.

A large microbial community exists in the human body, and typically microbes maintain a symbiotic relationship with the host; however, when the microbial ecosystem is out of balance, it may trigger the onset of host disease, including cancer [4, 9, 10, 11]. There is a growing body of evidence linking the human microbiome to cancer and cancer outcomes, including viruses, bacteria, and fungi [4, 12]. Due to the abundance of microorganisms present in the gut, previous studies have focused on the role between gut flora and tumors. With the development of applications of next‐generation sequencing technologies, we are beginning to identify bacterial DNA signatures within tumor cells [13, 14]. Current studies have shown that intratumor microbes promote tumor development mainly through DNA mutations, modulation of oncogenic pathways, and induction of chronic inflammation, and the impact of intratumor microbes on tumor chemotherapy and immunotherapy is gradually becoming a point of research. Studies have shown that the distribution of microorganisms within a tumor is not random, but a high degree of organization in a specific microecology known as the tumor microbe microenvironment. Microbiota play an important role in the tumor microbe microenvironment, influencing immune cell and epithelial cell function and thus contributing to cancer progression [15]. Notably, there is growing evidence for the presence and potential role of microbiota in the tumor microenvironment (TME) and its potential role. These intratumor microbes are tumor‐specific, and mechanistic studies have shown that tumor‐associated microbiota can influence cancer development, progression, and response to chemotherapy or immunotherapy [16, 17]. Studying the distribution and mechanisms of microbes in tumors will become a new research hotspot. In this review, we will focus on the tumor microbe microenvironment, intratumor fungi, and the use of hydrogels to intratumor fungal aspects, and explore potential new technologies and methods to influence intratumor microbes to control tumor development and metastasis.

## 2 The Tumor Microbe Microenvironment

The tumor microbe microenvironment differs from the commonly mentioned TME in that microorganisms including fungi, bacteria, viruses, and mycoplasmas may be present within tumor tissues [18]. It has been shown that microbial residues or metabolites have been observed within cancer cells and tumor‐infiltrating immune cells, which can accumulate within tumors and bind to receptors on cancer cells and immune cells, and that these microorganisms may influence the state of the TME and play a role in tumorigenesis, progression, metastasis, and immune response [19, 20, 21, 22].

There are three known possible sources of intratumor microbes, including mucosal barrier invasion, adjacent tissue invasion, and hematogenic invasion [23]. Studies have shown that microorganisms on mucosal surfaces may invade tumor tissues through damaged mucosa, and many intratumor microorganisms have been found to colonize mucosal organs such as the esophagus, lungs, colon, and cervix. Among them, intestinal and oral microbes are important sources of intratumor microbes [24, 25]. In addition, it has been found that intratumor microbiota in nonmucosal organs such as the pancreas may be translocated from the gut where the mucosal barrier is compromised, enter the pancreas through the pancreatic ducts, or be carried to the tumor site from disrupted blood vessels, where they colonize and multiply [26, 27].

Studies have shown that there is a specific aggregation of microorganisms in tumors for a variety of reasons. In addition to the “aboriginal” microorganisms in the tumor tissue, microorganisms from other organs or metastatic foci may also be transferred to the tumor tissue (adjacent tissue invasion). Currently, more attention and interests are focused on the study of intestinal flora. However, the study of microbiota in other sites is often neglected. Candida species and *Saccharomyces cerevisiae* are highly abundant in gastrointestinal (GI) tumor tract and contain highly similar flora in tumor and blood samples as determined and analyzed. This suggests that fungal DNA may be transferred from GI tumor sites into the bloodstream and circulate within the bloodstream of cancer patients, giving it promise as a predictive biomarker [28]. Several studies in recent years have shown that microorganisms, such as bacteria, are widely present in solid tumors and that they play a key role in the development and metastasis of cancer. The colorectal cancer (CRC) tumor‐resident bacteria *Escherichia coli* can disrupt the gut vascular barrier (GVB), after which the bacteria spreads to the liver, which may eventually lead to CRC metastasis to liver [29]. Thus, it is evident that intratumor microbes may be potential targets or tools for cancer prediction, treatment, and prognostic testing. Indeed, bacteria within tumors have been identified early on, but due to technical difficulties (e.g., lack of techniques to rule out the possibility of contamination), they have only begun to be widely recognized and explored in recent years. Microbiome analysis has been significantly improved due to the continuous advances in next‐generation sequencing [30]. Current methods for characterizing bacteria or fungi within the tumor microenvironment include PCR, microbial population identification sequencing (16S/18S/ITS), whole metagenome sequencing (WMS), immunohistochemistry (IHC), fluorescence in situ hybridization (FISH), and so on [31, 32] (Table 1).

**TABLE 1:** Some methods for characterizing bacteria or fungi within the tumor microenvironment.

Detection methods and techniques	Subject	Types of cancer	References
The two‐sided Wilcoxon test (bacterial 16S rRNA and fungal 28S rRNA expression) Immunohistochemistry (IHC)	The lung intratumor microbiome	Bacteria and fungi	[33]
Real‐time PCR targeting the 16S rRNA gene	Pancreatic ductal adenocarcinoma (PDAC):	Bacteria	[34]
16S rRNA sequencing Fluorescence in situ hybridization (FISH)	Colon cancer	Bacteria	[35]
16S rRNA sequencing	Lung cancer	Bacteria	[36]
Whole metagenome sequencing (WMS)	Colorectal cancer	Bacteria and fungi	[37]
Quantitative real‐time PCR targeting the 16S rRNA gene	Colorectal cancer	Bacteria	[38]
16S rRNA sequencing	Pancreatic cancer (PC)	Bacteria	[39]
16S rRNA next‐generation sequencing Quantitative PCR	Vulvar squamous cell carcinoma (VSCC)	Bacteria	[40]

**FIGURE 1:**
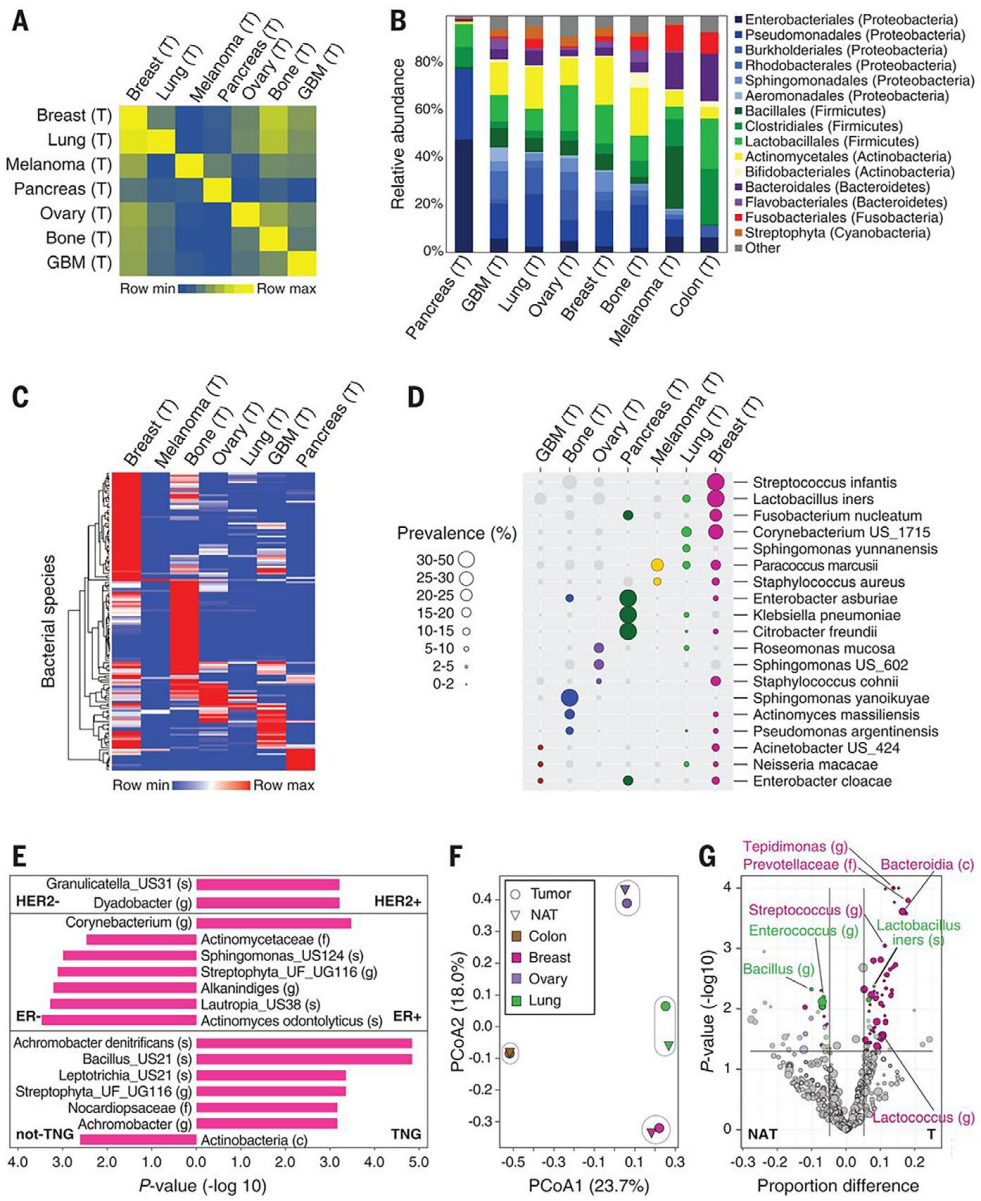
Different tumor types have distinct microbial compositions. (A) The heatmap displays the mean Jaccard similarity scores calculated from the bacterial species profiles of tumor samples (*n* = 528) that passed all filters, for all possible combinations of samples across different cancer types. (B) The arrangement of order‐level phylogenetic types is depicted across a range of tumor categories, with the relative frequencies being determined by the total count of reads for species that have cleared all the filters and are grouped within the same order. (C) An unsupervised hierarchical clustering approach was applied to the distribution patterns of 137 bacterial species, which were flagged as prevalent in one or more tumor types and detected in at least 10% of the samples within at least one of these tumor categories. (D) The distribution of 19 bacteria from category (C) is shown across a spectrum of tumor types, with only those bacteria that have been detected as hits in a particular tumor type being marked by colored circles. (E) A bar chart illustrates the significant differences in the prevalence of certain bacterial taxa among various subtypes of breast tumors. (F) The Principal Coordinate Analysis (PCoA) biplot depicts the relationships between the bacterial species compositions of different tissue types, based on Jaccard similarity indices. (G) A volcano plot is used to show the differences in bacterial prevalence between tumor (T) and normal adjacent tissue (NAT) samples in breast, lung, and ovary. P values are determined using a two‐sample proportion z test. Copyright 2020, The American Association for the Advancement of Science (AAAS).

Intratumor bacteria are predominantly intracellular and present in cancer cells and immune cells. Nejman et al. studied 1526 tumors and their adjacent normal tissues from seven cancer types, including breast, lung, ovarian, pancreatic, melanoma, bone, and brain tumors, and using detection techniques such as IHC, FISH, and 16S rDNA sequencing, the study revealed that there is a unique microbiome for each tumor type (Figure 1) [41]. It would be interesting to further explore the effects of intratumor bacteria on different phenotypes of cancer cells, the tumor immune system, and their interactions with tumor cells, regardless of whether they play a causal role in tumorigenesis and development.

Other side, the current correlative studies have shown that tumor tissue microbes are distinctly different from those of normal tissues. Where the basis for the presence of fungi in tumors is limited and needs to be further demonstrated, recent studies are illustrating the potential role of fungi within tumors, which will be the focus of this paper. Studies showed that the pathogenesis of pancreatic ductal adenocarcinoma (PDAC) may be related to fungi [42]. Using techniques such as 18S rRNA gene sequencing in PDA tumors of mice and humans, the researchers detected that the fungal content was about 3000 times greater than that of normal pancreatic tissue and was significantly enriched for Malassezia. At the same time, the researchers orally administered GFP‐labeled *Saccharomyces cerevisiae* to tumor‐bearing mice and detected the fungus entering the pancreas from the intestinal lumen, suggesting that the fungal mycobiome can directly influence the tumor microbe microenvironment. Furthermore, studies have demonstrated that immune tolerance could arise from the microbiota's reshaping of the pancreatic TME. Consequently, the integration of chemotherapy and immunotherapy with well‐targeted microbiota modulation might amplify the effectiveness of cancer therapies and enhance the survival outcomes for individuals with PDAC [43].

## 3 Intratumor Fungi

Fungal infections currently cause more than 1.5 million deaths worldwide each year [44]. Like bacteriome profiles, the variance in fungal composition among individuals within a population stems from a sophisticated interaction of multiple factors such as location, gender, age, ethnicity, diet, and lifestyle [45]. Intratumor fungal communities are an important part of the TME, and their interactions with the host are critical for cancer development and progression. Recent studies have found that fungi are not only present in a wide range of cancer types, but that their abundance and diversity differ markedly between cancer types. In head and neck cancer, the presence of *Candida* and *Saccharomyces* correlates with similar types of bacteria. Notably, strains of *Lactobacillus*, especially *Lactobacillus gasseri* , are often found in association with *Candida*, but are less commonly found with *Saccharomyces* [28]. 2022 Straussman's scientific team conducted a study of 35 cancers and their associated fungi, creating the first pan‐cancer fungal biome atlas, and most of these fungi have been found to be present within the tumor cells or in the immune cells within the tumor. Using Weizmann [WIS], the team first tested the relationship between fungi and disease phenotypes, patient survival, and treatment response [46]. In breast cancer, the researchers found that *Cladosporium sphaerospermum* and *Cladosporium genus*, previously reported in breast cancer, were enriched in tumors of patients ≥ 50 years old [46, 47]. In lung cancer, fungal abundance and enrichment of Aspergillus and Agaricomycetes were higher in tumors of current smokers compared to nonsmokers. In addition, Liu et al. identified enriched tumor‐resident *Aspergillus sydowii* in lung adenocarcinoma (LUAD) patients using fungal‐enriched DNA extraction and deep shotgun macrogenome sequencing, which promotes the progression of lung cancer despite the low biomass of the intratumor fungal group and can be strain‐level targeted to improve the prognosis of LUAD patients. The mechanism by which Aspergillus sydowii induces a microenvironment of tumor immunosuppression and ultimately promotes the development of lung cancer through interactions with the host was elucidated [48].

In addition, some fungi seem to be able to manipulate the immune system to benefit tumors, similarly to bacteria. A fungus called *Malassezia globosa* has been found to promote the development of pancreatic cancer and has also been found in breast cancer patients, who tend to have shorter overall survival. Additional studies have noted that some fungi in pancreatic cancer intercept part of the immune system, which promotes tumor growth. Also, in the early stages of pancreatic tumorigenesis, when massive oxidative stress is not detected in pancreatic intraepithelial neoplasia, fungal components can be detected [42, 49, 50]. A study for 2022 by Cornell University microbiologist Iliyan Iliev and his team showed that expression levels of proinflammatory genes were elevated in Candida‐rich gastric cancer tissues and that Candida DNA‐rich colon tumors were more likely to metastasize [28]. There is an interaction between fungi and the host immune system, which is how fungi can affect cancer by modulating the host's immune response. The host innate immune system can recognize microbe‐associated molecular patterns (MAMPs) on the fungal cell wall through pattern recognition receptors (PRRs). These receptors include C‐type lectin receptors (be like Dectin‐1), Toll‐like receptors, and Nod‐like receptors (NLRs) [51]. When fungal cell wall components bind to PRRs on the host cell surface, signaling is triggered; for example, Fungi produce signaling molecules such as interleukin 1β (IL‐1β), IL‐6, IL‐12, IL‐23, TGF‐β, and interferon gamma (IFN‐γ) through pathways such as SYK‐CARD9, SYK‐PLCγ2, MYD88, or TRIF. Activation of related signaling pathways further promotes the immune response of T helper 1 (Th1) and Th17 cells, which play a role in inflammation and immune surveillance [52]. Meanwhile, fungi may promote tumor development by affecting the TME, for example, by altering metabolic pathways in tumor cells or by promoting the accumulation of immunosuppressive cells [53]. Bacteria and fungi may also form a biological barrier known as a biofilm, which can protect the microorganisms from the host's immune system and potentially worsen local inflammation. For instance, the fungal pathogen *Candida albicans* can create polymicrobial biofilms in conjunction with *Actinomyces naeslundii* and *Streptococcus mutans* , leading to the malignant transformation of oral keratinocytes. Additionally, the secretions from these biofilms facilitate the adhesion of oral squamous carcinoma cells to the extracellular matrix and induce the production of proinflammatory cytokines, such as IL‐6 [54, 55].

Fungi are known to have complex metabolism. Fungal‐modulated metabolites may affect the host, and these fungal‐associated bioactive metabolites are thought to promote tumorigenesis and progression through multiple mechanisms [56]. The current study recognizes the increased detection of Candida in patients with oral cancer and the metabolism of Candida to produce the carcinogens acetaldehyde and nitrosamines. Candida secretes ethanol dehydrogenase, which metabolizes ethanol into carcinogenic acetaldehyde, which is involved in the pathogenesis of oral cancer [57, 58, 59]. Other fungal carcinogens include *Aspergillus, Pencillium, Paecilomyces*, and *Byssochlamys species* [60]. It is worth noting that these carcinogen‐secreting fungi described above, and a variety of other fungi that have not been identified to date, are not considered commensal for humans, yet humans can be exposed to them or their potential carcinogenic products through environmental and food‐related pathways. Therefore, strain differences in carcinogenic potential, host susceptibility factors, confounding factors associated spuriously driving, and other complex factors with fungal cancer‐modulating effects deserve further investigation [61, 62].

**FIGURE 2:**
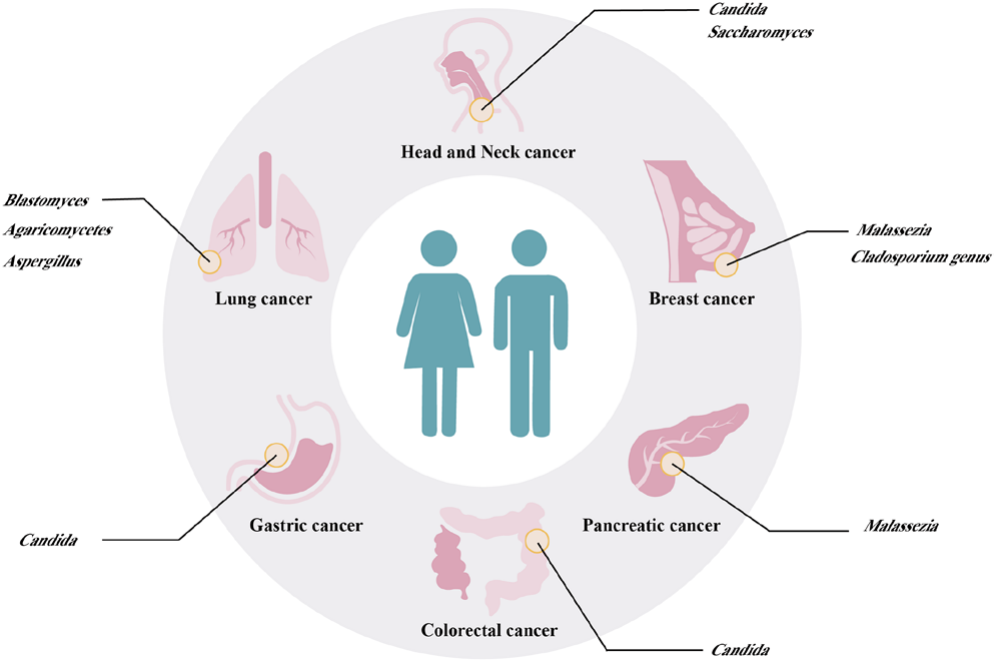
Known distinct mycobiome signature in various types of cancer. It shows that mycobiome signatures differ among cancer types. Changes in tumor‐associated microbial communities, a key part of the tumor microbiome, may affect the tumor immune environment and influence tumor progression.

Although the biomass of fungal communities within tumors is low, they play an important role in stimulating the immunosuppressive TME and are strongly associated with poor patient prognosis. These findings may provide novel fungal markers for early cancer screening and new ideas for personalized cancer treatment strategies based on fungal targeting. The researchers have pinpointed a distinctive pattern of circulating fungal DNA from 20 different fungi, which could serve as a reliable indicator to differentiate between individuals with various cancers and those who are healthy. This finding highlights the significant potential of utilizing the mycobiome for cancer diagnosis, making it a valuable tool even for patients at the early stages of cancer [63]. As shown in Figure 2, recognized mycobiome signatures vary across different types of cancer. Meanwhile, changes in the tumor‐associated microbial communities, which are “key group” in the tumor microbiome, may have an impact on the tumor immune environment, thus influencing tumor development and progression.

## 4 The Unique Advantages of Antifungal Hydrogels Compared to Traditional Antifungal Therapies

### 4.1 Targeting and Localized Therapy Advantages

Hydrogels can be precisely engineered to deliver antifungal agents directly to the site of fungal infection, offering targeted treatment. Unlike conventional antifungal drugs, which require systemic administration to achieve effective concentrations, hydrogels can reduce the distribution of drugs throughout the body, thereby minimizing toxicity to healthy tissues. For instance, Kai et al. [64] investigated a cisplatin‐loaded hydrogel nanoparticle (PRINT‐Platin), examining its drug payload, pharmacokinetics, and toxicity, as well as its potential in cancer therapy. Their findings indicated that PRINT‐Platin accumulated at least twice as much in tumors compared to cisplatin alone, demonstrating that the nanoparticle formulation could more effectively deliver the drug to the tumor site. Additionally, PRINT‐Platin exhibited lower cytotoxicity and reduced accumulation in the kidneys, suggesting a lower potential for nephrotoxicity. Moreover, hydrogels can be functionalized through chemical modification or physical methods to achieve targeted drug delivery. For example, Farjadian et al. [65] developed a thermosensitive and pH‐responsive nanohydrogel drug delivery system based on lysine‐modified poly (vinyl caprolactam); Ghasemi et al. [66] designed a spermine‐modified PNIPAAm nanohydrogel as a thermosensitive system for cisplatin delivery; and Lin et al. [67] enhanced and controlled transdermal drug delivery using erbium laser technology, further exploring the delivery of lipophilic and hydrophilic drugs. Such targeting is challenging to achieve with traditional antifungal therapies.

### 4.2 Multifaceted Mechanisms and Synergistic Effects

Hydrogels can integrate multiple functionalities, and their multifaceted mechanisms and synergistic effects can be harnessed for antifungal treatment. These mechanisms include physical barrier formation, controlled release of antimicrobial agents, photothermal effects, quorum sensing inhibition, immune regulation, and anti‐inflammatory actions. For instance, the BEPE hydrogel achieves highly efficient inhibition of *Candida albicans* by combining photothermal effects, the antimicrobial action of ε‐polylysine, and the quorum sensing inhibition of epigallocatechin gallate (EGCG). This integration encompasses several mechanisms such as photothermal ablation, release of antimicrobial components, and quorum sensing inhibition [68]. Moreover, nanocomposite hydrogels, which combine the dual advantages of hydrogels and nanozymes, possess multiple antifungal mechanisms and dual tissue repair mechanisms. They demonstrate higher synergistic effects and therapeutic outcomes compared to traditional antifungal drugs [69].

### 4.3 Addressing Drug Resistance and Antifungal Toxicity

Traditional antifungal drugs may exhibit toxicity, and their overuse can lead to increased drug resistance. In contrast, hydrogels can be intelligently designed to reduce the dosage and frequency of drug administration. For instance, the emergence of a fungal enzyme‐responsive hydrogel that enables triggered release of antifungal drugs can respond to the secretion of aspartic proteases (Saps) by the toxic *Candida albicans* . This hydrogel releases the drug only at the site of infection, where it is degraded by Saps, thereby minimizing unnecessary drug exposure and reducing the risk of resistance development [70].

### 4.4 Cost‐Effectiveness

(1) Reducing Costs through Materials and Technology: Fungal infections, such as cryptococcosis, often face challenges of limited treatment options, high recurrence rates, and high treatment costs. The main components of hydrogels are typically natural or synthetic polymers, such as hyaluronic acid and chitosan, which are widely available and low cost. Moreover, hydrogels can be combined with low‐cost antimicrobial drugs through 3D printing technology, further reducing treatment costs [71]; (2) Reducing Drug Dosage to Minimize Toxicity: Hydrogels can locally release drugs, reducing systemic exposure and thereby decreasing drug dosage and potential side effects. For example, nanocomposite hydrogels achieve targeted release of antifungal drugs, reducing drug usage, resistance risks, and potential nanotoxicity [72]; (3) Shortening Treatment Duration and Reducing Complications: Studies have shown that the biocompatibility and tissue repair‐promoting properties of hydrogels can shorten treatment duration and reduce complications, indirectly lowering medical costs. In the research on antifungal hydrogels, Bhattacharjee et al. [73] developed a water‐soluble quaternized polyethyleneimine derivative (QPEINH‐C6) and combined it with biocompatible gellan and polyvinyl alcohol (PVA) to create a novel antimicrobial hydrogel. This hydrogel exhibits rapid bactericidal activity against various drug‐resistant bacteria and fungi, significantly reducing microbial load in a short time and thus shortening the treatment duration. Additionally, it shows good antibiofilm activity against preformed bacterial biofilms and polymicrobial biofilms, helping to reduce complications associated with biofilms.

### 4.5 Feasibility of Clinical Application

(1) High Biocompatibility and Safety: Traditional hydrogels containing antibiotics or metallic nanoparticles often induce bacterial resistance, exhibit low biocompatibility, and lack real‐time monitoring capabilities. In contrast, hydrogels possess excellent biocompatibility and biodegradability, reducing tissue irritation and toxicity, making them suitable for long‐term use. For instance, Cui et al. [74] developed a fluorescent antimicrobial hydrogel with antibacterial capabilities, superior optical properties, and high biocompatibility. This hydrogel demonstrates antibacterial performance 108.5 times higher than that of other similar hydrogels. No significant cytotoxicity or tissue toxicity was observed after implantation or co‐incubation with cells, indicating its high safety profile; (2) Strong Adaptability: Hydrogels can be customized to meet diverse clinical needs. For example, nanocomposite hydrogels for treating fungal keratitis not only exhibit antifungal properties but also promote corneal repair. Sha et al. [75] developed a novel triblock polymer nanoparticle–gel complex (Gel@PLGA‐PEI‐PEG@NAT) for delivering natamycin, enhancing its efficacy in treating fungal keratitis.

## 5 Current Challenges in Oncology and the Application of Hydrogels in Cancer Treatment

Current conventional tumor treatments, such as chemotherapy, are effective to a certain extent, but their serious adverse effects have led to limitations in the application of the drug. The lower therapeutic index and poorer targeting of chemotherapeutic drugs make it difficult not to produce toxicity during the treatment process, and they cannot act precisely on the tumor cells, leading to the damage of normal cells. Also, long‐term use of chemotherapeutic drugs may have led to the production of tolerance in tumor cells. The above drawbacks have prompted researchers to look for new cancer treatment strategies [76, 77].

As the methods and means of treating cancer are constantly being updated, compared with systemic administration, local continuous administration at the tumor site or tumor resection site will become one of the effective means of tumor treatment, especially for inhibiting tumor recurrence. Hydrogels or nanogels have a three‐dimensional network structure and are materials with stable chemical properties, low toxicity, good biocompatibility, and biodegradability, and they can be widely used as drug carriers [78]. They are closer to natural active tissues than other synthetic biomaterials because they possess high water absorption, porosity, and a certain degree of soft consistency [79]. Due to their special structure and properties, they have been widely used in drug delivery. Studies have shown that hydrogels have a high drug loading capacity, which enhances the efficiency of cellular uptake, as well as the ability to protect the drug and delay its degradation, in terms of drug‐targeted delivery, and are therefore highly promising for research [80, 81].

Recent studies have shown that the main application of hydrogels in oncology therapy is as a drug delivery platform, especially for targeting and immunotherapy of microorganisms in tumors. With the rapid development of immunotherapy in the field of tumor treatment, patients may be affected by factors such as drug efficacy, drug retention time, and systemic adverse effects, and the response to immunotherapy is low [82]. Injectable hydrogel systems, as drug carriers, have the advantages of easy application, increased local drug concentration, prolonged drug retention time, and good biocompatibility. They have especially attracted much attention as a drug delivery system for local immunotherapy or immunocombination chemotherapy [83]. Injectable hydrogels can deliver immune cells (e.g., CAR‐T cells), immune checkpoint inhibitors (e.g., PD‐1, PD‐L1, and CTLA‐4), and cytokines [84, 85, 86].

**FIGURE 3:**
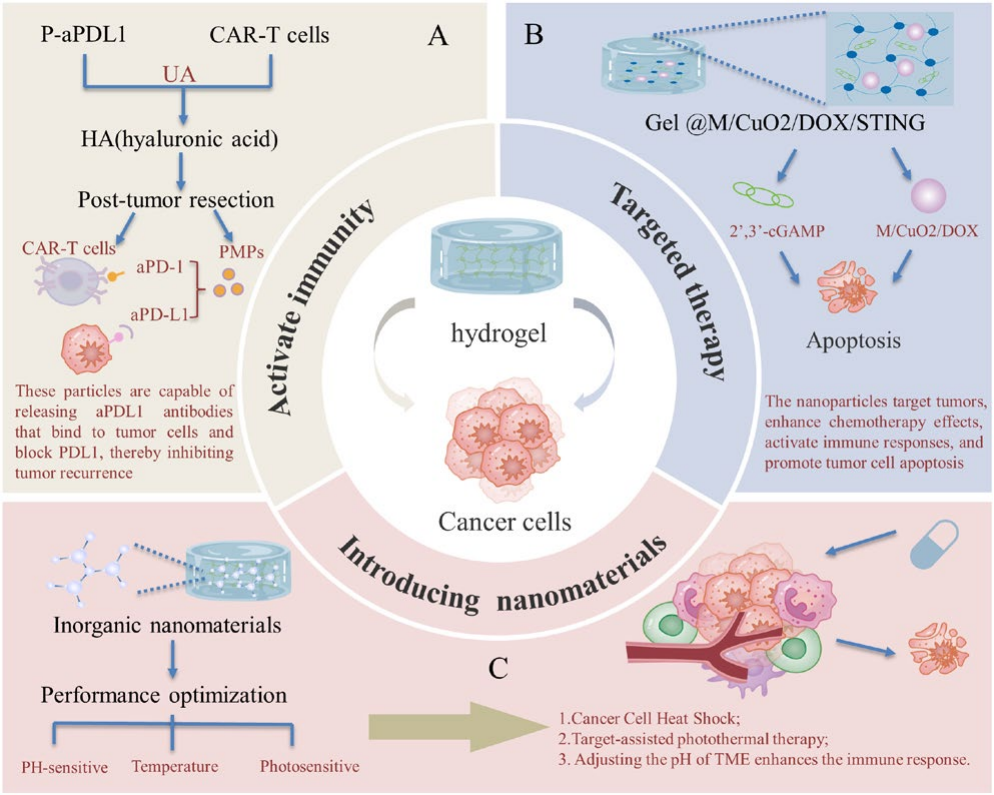
Hydrogels in tumor therapy. (A) A novel hyaluronic acid‐based hydrogel can load CAR‐T cells and P‐aPDL1 platelets for local treatment after tumor resection. It serves as a cell reservoir and activates platelets to form PMPs in the postoperative inflammatory environment, releasing aPDL1 antibodies to block PDL1 and inhibit tumor recurrence. (B) An injectable zwitterionic physical hydrogel encapsulating M/CuO2/DOX nanoparticles and STING agonist 2',3'‐cGAMP, which targets tumors to enhance DOX uptake and synergistic therapy effects, releases STING agonist to activate the STING pathway and remodel the TME, and then targets residual tumor cells to induce DNA damage and immunogenic cell death. (C) By incorporating inorganic nanomaterials with specific functionalities into hydrogels, it is possible to significantly expand the application range of hydrogels, endow them with new properties, and enhance their responsiveness to external stimuli. For example, changing the pH of chemotherapy drugs can improve their efficacy, photothermal therapy can be used as an adjuvant for cancer treatment, and thermosensitive hydrogels, as an advanced drug delivery platform, can release antitumor drugs on demand and effectively penetrate tissues, effectively addressing the problems of drug resistance and side effects associated with traditional cancer therapy.

While CAR‐T therapy has achieved several successes in treating tumors since 2017, significant breakthroughs in the treatment of solid tumors remain elusive. In 2022, Grosskopf and colleagues developed an innovative approach by incorporating CAR‐T cells and cytokines into a specially formulated hydrogel. This hydrogel serves as a temporary, in vivo environment that activates the immune cells. Once activated, the CAR‐T cells are then directed to target and attack tumor tissues, demonstrating promising potential for the treatment of distant tumors [84]. In 2024, Zhu et al. [87] reported for the first time the development of an injectable supramolecular hydrogel system that enables in situ reprogramming of CAR‐T cells by loading plasmid CAR (pCAR). In humanized mouse models, this system successfully achieved in situ generation of CAR‐T cells and their effective accumulation at the tumor site. Moreover, by reprogramming the tumor microenvironment, the system significantly enhanced the secretion of cellular inflammatory cytokines and tumor‐killing proteins, thereby reversing the immunosuppressive microenvironment and promoting the infiltration of CAR‐T cells and cytotoxic T cells within the tumor. Moreover, researchers have developed a novel hyaluronic acid (HA)‐based hydrogel capable of loading CAR‐T cells and anti‐PDL1 blocking antibody‐bound human platelets (P‐aPDL1). This hydrogel is formed by cross‐linking under UV light irradiation and is used for local treatment after tumor removal. The hydrogel not only serves as a reservoir for CAR‐T cells and platelets, but also triggers platelet activation in the postsurgical inflammatory milieu, leading to the formation of platelet‐derived particles (PMPs). These particles are capable of releasing aPDL1 antibodies that bind to tumor cells and block PDL1, thereby inhibiting tumor recurrence (Figure 3A) [88].

In addition to this, the hydrogel can be designed to target and participate in tumor treatment from multiple aspects. Below et al. designed polyethylene glycol (PEG)‐based hydrogel scaffolds to mimic PDAC organoid, helping to better understand the interactions between tumor cells and the surrounding stroma, and how these interactions affect tumor progression and response to treatment [89]. The lncRNAs have been shown to regulate autophagy and, due to the dual nature of autophagy, dictate the dual regulatory role of Autophagy‐related lncRNAs, affecting tumor development and drug resistance [90]. Already, hydrogels loaded with RNA have shown great promise for disease intervention at the gene level, and some of these therapies have been approved for clinical use. Unlike molecular or nanoscale approaches, macroscopic hydrogels are soft, water‐swellable, three‐dimensional structures with remarkable features such as biodegradability, modifiable physicochemical properties, and injectability. Such hydrogel systems are not only capable of sustained local delivery of RNA (avoiding repeated dosing) but also of spatial and temporal control of the release rate [91]. Designing a hydrogel that can adhere to tumor cells by interacting with the cell membrane's reactive thiol‐enriched interface can impede the cells' ability to move invasively. This approach, when paired with chemotherapy postbrain tumor surgery, presents a potential new strategy for combating glioblastoma by suppressing both the invasiveness and the growth of residual cancer cells [92]. Fang et al. designed an innovative injectable zwitterionic physical hydrogel system that encapsulates M/CuO_2_/DOX nanoparticles and the interferon gene stimulator (STING) agonist 2′,3′‐cGAMP, collectively referred to as Gel@M/CuO_2_/DOX/STING. These M/CuO_2_/DOX nanoparticles possess tumor‐targeting capabilities, enhancing the uptake of the drug Doxorubicin (DOX) by tumor cells and amplifying the synergistic effects of chemotherapy and chemodynamic therapies. Following this, the STING agonist is released from the hydrogel through a sustained release mechanism, which activates the STING pathway and remodels the immunosuppressive TME. Subsequently, M/CuO_2_/DOX nanoparticles target residual tumor cells, inducing DOX‐induced DNA damage, immunogenic tumor cell death, and copper‐induced cell death (Figure 3B) [93].

On the other hand, conventional hydrogels are typically formed by connecting polymers through physical or chemical means, a method that has a limited effect on enhancing the properties of hydrogels. However, by incorporating inorganic nanomaterials with specific functionalities into hydrogels, it is possible to significantly broaden the range of applications for hydrogels and endow them with a variety of new properties, as well as to markedly enhance their responsiveness to external stimuli [94, 95]. The acidic substances produced by cancer cell metabolism can cause a decrease in the pH value of the TME. This acidic environment enhances the metastatic phenotype of cancer cells and their resistance to chemotherapy drugs. Research has shown that adjusting the pH of the TME can boost the immune response [96, 97, 98, 99, 100]. The Chitosan‐Poly (ethylene glycol) (CS‐PEG) hydrogel can improve the efficacy of the weak base chemotherapy DOX. The CS‐PEG hydrogel offers alkaline buffering by releasing sodium bicarbonate to neutralize the acidic conditions, increasing the release of DOX in a low pH environment, and decreasing it in a high pH environment [101]. Photothermal therapy for adjuvant cancer treatment involves the use of specific photosensitizers that generate heat under the irradiation of a light source, such as a laser, to raise the temperature of the tumor tissue to 40°C–43°C. This temperature range is sufficient to inflict heat shock on cancer cells, thereby directly killing them or inhibiting their proliferative capacity [102, 103, 104]. As an advanced drug delivery platform, thermosensitive hydrogels can release antitumor drugs on demand and effectively penetrate tissues, effectively addressing issues of drug resistance and side effects associated with conventional cancer therapy [105, 106]. All these new ideas and applications are reflected in Figure 3C.

In conclusion, hydrogels offer significant advantages in the treatment of tumors, have a broad application prospect, and are anticipated to be an effective therapeutic strategy.

## 6 Potential Utilization of Hydrogels to Combat Fungi Within Tumors

### 6.1 Applications of Hydrogels in Microbe‐Tumor Modulation

Hydrogel formulations hold broad application prospects in the modulation of tumor microbiota. They can enhance antitumor immune responses and inhibit tumor growth and metastasis by delivering microbe‐derived immunomodulators (such as outer membrane vesicles, OMVs), reshaping the tumor microenvironment, and achieving controlled and sustained drug release. OMVs have great potential in tumor immunotherapy, as they can activate the immune system and suppress tumor growth. For instance, a smart DNA hydrogel that specifically captures OMVs and combines with PD‐1 aptamers to block immune checkpoints has achieved highly efficient local immunotherapy. In a mouse model of melanoma, the tumor inhibition rate of this hydrogel can reach approximately 95% [107].

Hydrogels can also modulate the tumor microenvironment through various mechanisms, shifting it from an immunosuppressive to an immunostimulatory state. A macrophage and DC (Dendritic Cell)‐boosting hydrogel (TCCaGM) was meticulously engineered by encapsulating granulocyte–macrophage colony‐stimulating factor (GM‐CSF) and a therapeutic nanoplatform (TCCaN) preloaded with tunicamycin (Tuni) and catalase (CAT), with the assistance of calcium carbonate nanoparticles (CaCO_3_ NPs). The TCCaGM hydrogel reshaped the tumor microenvironment (TME) by upregulating calreticulin (CRT) and downregulating CD47, thereby enhancing the antitumor activity of macrophages and dendritic cells (DCs). It also demonstrated robust bioactivity in the 4T1 breast tumor model. Furthermore, the combination of TCCaGM with PD‐1 antibody (αPD‐1) treatment significantly amplified the antitumor efficacy, markedly inhibiting the growth of both primary and distant tumors and prolonging survival [108].

As a drug carrier, hydrogels can achieve controlled and sustained release of chemotherapeutic drugs, thereby enhancing the local concentration of drugs at the tumor site and reducing systemic toxicity. For example, an injectable hydrogel based on hyaluronic acid can achieve local drug release through dynamic covalent bonds and deliver the chemotherapeutic drug cisplatin via double‐stranded DNA [109]. Cisplatin not only acts as a chemotherapeutic agent but also serves as a catalyst for chemodynamic therapy (CDT), initiating a cascade reaction through the Fenton reaction. This design improves local drug efficacy, reduces systemic toxicity, and lowers the risk of drug resistance.

Moreover, hydrogels can combine microbial agents and immunomodulators to achieve synergistic therapeutic effects. For instance, targeting the highly immunosuppressive tumor microenvironment of pancreatic cancer, a novel BV/TP5 hydrogel was developed through co‐assembly. It significantly enhances the activity of immune cells such as CD4+ and CD8+, effectively inhibits the in situ growth of pancreatic cancer cells, and stimulates the body to mount an immune response, thereby suppressing the emergence of distant metastases [110].

### 6.2 Design and Advantages of Fungus‐Regulating Hydrogels in Cancer Therapy

To further investigate the functions and interactions of fungi, as well as their relationship with tumor development and treatment response, researchers have developed various approaches. Through these methods, they can better understand the mechanisms of fungi in cancer and provide new ideas and strategies for cancer prevention and treatment. Hydrogel, as a topical treatment, may have a potential impact on regulating the intratumor microbes by altering the activity of immune cells in the TME; hydrogel may indirectly affect the composition and function of intratumor flora. As a biomaterial, hydrogel's application in oncology therapy is not limited to drug delivery, but also as an enhancement platform for immunotherapy, which may help to improve the targeting of intratumor flora. According to mucosal barrier invasion, one of the previously mentioned causes of intratumoral microbial origin, hydrogels may modulate the flora at the source, thereby affecting tumorigenesis and progression. It has been shown that oral digestion of Peptostreptococcus in patients with oral squamous cell carcinoma (OSCC) activates the immune system and improves the prognosis of the patients, induces anti‐cancer immune responses, and inhibits tumor growth. Preparation of hydrogels with silver nanoparticles (AgNP) allowed Peptostreptococcus to proliferate while inhibiting the growth of competing bacteria, and the results of the study suggest that biomaterials can be engineered to modulate the human microbiota to enhance antitumor immune responses [111].

The various effects of intratumor fungi have been described previously, and in terms of therapeutic approaches for the treatment of intratumor fungi, hydrogels, as a novel biomaterial, show great potential. By specific design, hydrogels can be targeted to treat fungi within tumors, for example, by combining antifungal drugs with hydrogels to achieve local release of the drug, thereby improving efficacy and reducing systemic side effects. In addition, the three‐dimensional network structure of hydrogels can be used as a drug delivery system to efficiently encapsulate drug molecules and enable controlled release of drugs through stimulus response, such as pH‐sensitive, temperature‐sensitive, or enzyme‐sensitive hydrogels [112]. The application of hydrogels has also shown advantages in terms of antifungal resistance. As fungal infections are more common in oncology patients and the problem of drug resistance is becoming more serious, especially in patients treated with cancer chemotherapy or using immunosuppressive therapy, they have a low‐functioning immune system, and they are more prone to develop resistance to fungal therapy. Hydrogels can be used as a novel antimicrobial carrier to reduce the occurrence of drug resistance by loading antifungal drugs or antifungal peptides that act directly on fungi within the tumor [113]. In addition, hydrogels can be combined with immunotherapy to enhance the effectiveness of tumor treatment. For example, tumor antigens and adjuvants can be combined into hydrogels, which can be injected locally into the tumor tissue to activate the immune system to attack the tumor [114]. In addition, hydrogels can serve as scaffolds for immune cells, promoting their infiltration and activity in the TME. In the treatment of intratumor fungi, the application of hydrogels is not limited to drug delivery but also includes early detection and monitoring as biosensors. For example, researchers have developed a locally embedded photodynamic immunomodulatory DNA hydrogel for early warning and inhibition of tumor recurrence after surgery. This hydrogel contains a PDL1 aptamer that traps and enriches in situ recurrent tumor cells, increasing local ATP concentrations to provide timely early warning signals [115]. The application of hydrogels in the treatment of intratumor fungi has demonstrated multifaceted potential, including as drug delivery systems, adjuvant materials for immunotherapy, and biosensors. However, hydrogels still require further research and development before clinical application to ensure their safety, effectiveness, and controllability.

**FIGURE 4:**
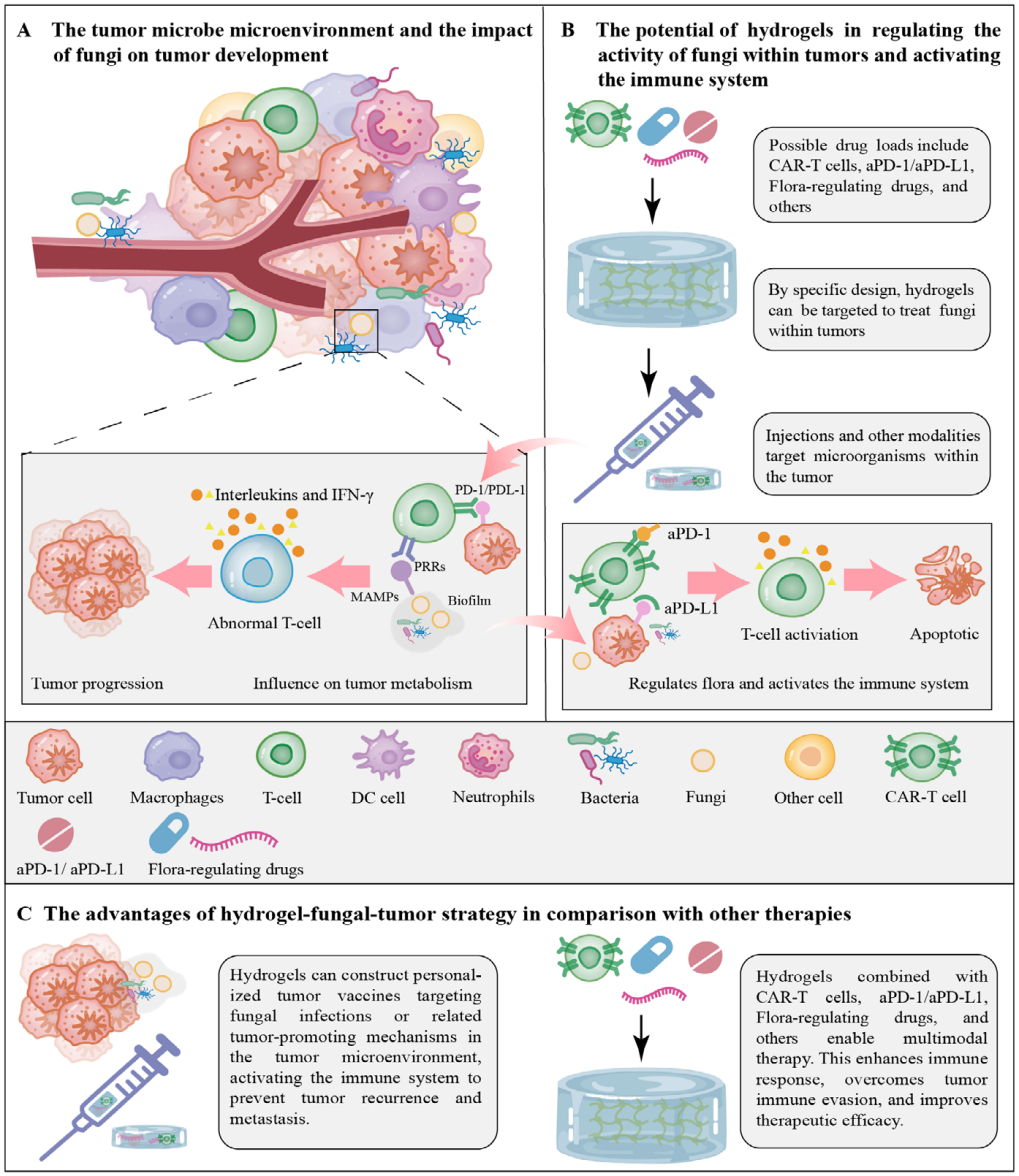
Perspectives on the impact of hydrogel application on fungal influences within the microbial microenvironment of tumors. (A) The tumor microbe microenvironment and the impact of fungi on tumor development. (B) The potential of hydrogels in regulating the activity of fungi within tumors and activating the immune system. (C) The advantages of the hydrogel–fungal–tumor strategy in comparison with other therapies.

In summary, hydrogels have a variety of potential applications in cancer therapy, particularly in therapeutic strategies related to tumor microbes. Future research may further investigate the use of hydrogels in regulating intratumoral fungi and enhancing immune responses to therapy. The mechanism by which hydrogels applied to regulate intratumoral fungi activate the immune system is illustrated in Figure 4.

## 7 Conclusions

The presence of fungi within the tumor is one of the causes of dysbiosis in the tumor microbial environment, and the use of immunotherapy in combination with biomaterials may overcome certain clinical barriers. Currently, both the fields of biomaterials development and cancer immunotherapy are growing at a phenomenal rate, with new materials and clinical trials appearing every year. While these combination therapies have improved efficacy, many side effects or toxicities have also emerged. Hydrogels have great advantages as drug delivery platforms, and together with related drugs, they play the roles of drug delivery, immunomodulation, and local therapy in the treatment of intratumoral fungi. They can be engineered to provide controlled release of drugs, immunomodulation to bolster the immune response, and localized therapy to directly address the fungal presence within tumors. The versatility of hydrogels allows for the encapsulation of a variety of agents, including but not limited to antifungal drugs, cytokines, and immune checkpoint inhibitors, which can work in concert to combat the fungal dysbiosis and tumor growth.

In this review, we can see that antimicrobial hydrogels are used in different ways for the treatment or monitoring of intratumoral fungi. As we advance in this field, ongoing research is dedicated to refining hydrogel technology, aiming to create more sophisticated and efficacious materials tailored for clinical use. The goal is to develop hydrogels that not only deliver therapeutic payloads effectively but also possess biocompatibility, biodegradability, and the ability to respond dynamically to the tumor microenvironment (TME), thereby improving patient outcomes and safety in cancer treatment.

Looking ahead, the development of hydrogels that can actively modulate the antitumor immune response and integrate with precision medicine approaches holds great promise. These advanced hydrogels could potentially reshape the tumor microenvironment, enhance immune surveillance, and provide a more favorable setting for the eradication of both fungi and tumor cells.

In conclusion, hydrogels show great potential and multifaceted advantages in the treatment of fungi within tumors to inhibit tumor progression. However, more research is needed to optimize these systems and conduct clinical trials to validate their safety and efficacy in humans. This will also provide a deeper insight into the remarkable contribution of fungi and their metabolites to the investigation of cancer and its treatment. We will continue to improve the hydrogel drug carrier technology to develop more and better novel materials for the clinical treatment of tumors.

## Author Contributions

P.C. and W.T. the main author of the study, conceived the study and contributed to writing and editing. A.Z., H.G., and J.Z. reviewed and revised the manuscript. All the authors read and approved the final manuscript.

## Conflicts of Interest

The authors declare no conflicts of interest.

## Data Availability

Data sharing is not applicable to this article as no new data were created or analyzed in this study.
